# Atypical Hemolytic Uremic Syndrome After Liver Transplant Treated With Eculizumab

**DOI:** 10.7759/cureus.9230

**Published:** 2020-07-16

**Authors:** Sundus Bhatti, Mohammed Alghamdi

**Affiliations:** 1 Internal Medicine, University of Louisville School of Medicine, Louisville, USA; 2 Pathology, University of Louisville School of Medicine, Louisville, USA

**Keywords:** eculizumab, calcineurin inhibitor, atypical hemolytic uremic syndrome, transplant

## Abstract

Thrombotic microangiopathies (TMA) are a group of serious conditions that are characterized by microangiopathic hemolytic anemia and thrombocytopenia, and are often associated with acute kidney injury as well as neurologic abnormalities. There are multiple causes of TMA. TMA in a transplant patient is often attributed to calcineurin inhibitor (CNI) use and is usually treated with discontinuation of the drug. We report a case of TMA in a liver transplant patient who did not respond to CNI discontinuation or plasmapheresis but had great response to shorter than usual course of eculizumab. Eculizumab is a monoclonal antibody that prevents c5 cleavage and indirectly inhibits the formation of the membrane attack complex. Clinical response was sustained for nine months after discontinuation of eculizumab.

## Introduction

Thrombotic microangiopathies (TMA) are a group of serious conditions that are characterized by microangiopathic hemolytic anemia and thrombocytopenia, and are often associated with acute kidney injury. There are multiple causes of TMA: ADAMTS-13 deficiency causing thrombotic thrombocytopenic purpura (TTP), Shiga toxin causing hemolytic uremic syndrome (HUS), different drugs causing TMA through immune-mediated mechanism or toxicity-mediated mechanism and complement activation disturbances causing atypical hemolytic uremic syndrome (aHUS). TMA in a transplant patient is often attributed to calcineurin inhibitor (CNI) use and is usually treated with discontinuation of the drug. Plasma exchange is used with variable success [1].

We report a case of TMA in a liver transplant patient who did not respond to CNI discontinuation or plasmapheresis but had great response to shorter than usual course of eculizumab. Eculizumab is a monoclonal antibody that prevents c5 cleavage and indirectly inhibits the formation of the membrane attack complex [1].

## Case presentation

A 56-year-old African American female with a past medical history notable for hepatitis C (treated and cleared) presented with acute liver failure secondary to accidental acetaminophen overdose. Workup for liver failure was sent, and her hepatitis panel, human immunodeficiency virus (HIV), cytomegalovirus (CMV) and herpes simplex virus (HSV) tests were negative. Ultrasound of the abdomen with Doppler showed no evidence of cirrhosis, masses and biliary dilation and demonstrated normal flow in the hepatic vessels.

**Figure 1:**
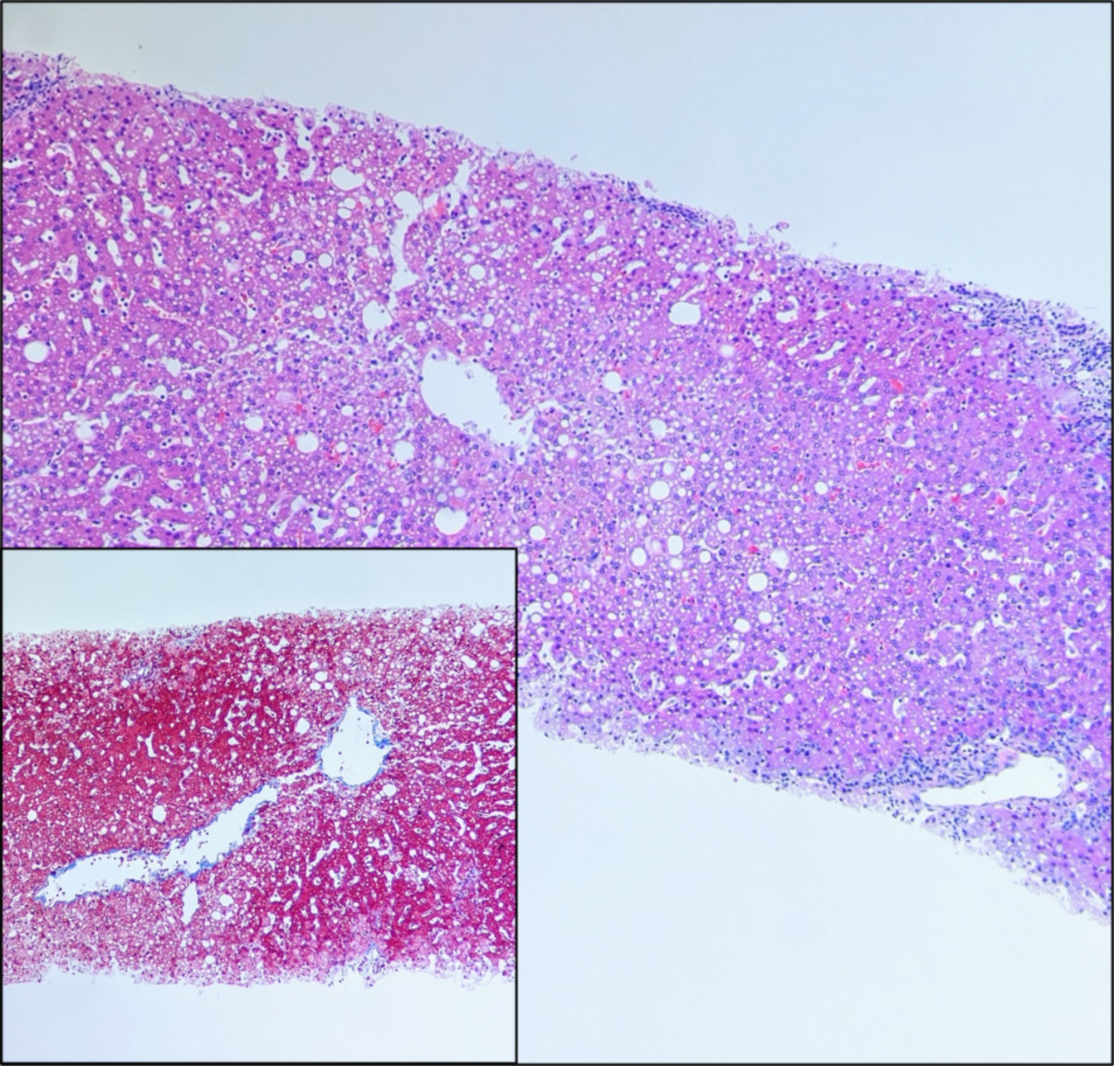
Photomicrographs of liver parenchyma showing sinusoidal dilation with micro- and macrosteatosis, features of preservation injury (hematoxylin-eosin stain, ×40). Inset shows the trichrome special stain highlighting the lack of fibrosis (trichrome stain, ×200).

She underwent an orthotropic liver transplant due to her acute liver failure. She was given induction chemotherapy with methylprednisolone and mycophenolate and was maintained on tacrolimus, mycophenolate and prednisone. Her liver function tests (LFTs) improved to baseline post-transplant. The histologic examination of the donor liver biopsy showed mild micro- and macrovesicular steatosis and centrilobular ischemic change, consistent with preservation injury (Figure 1).

**Figure 2:**
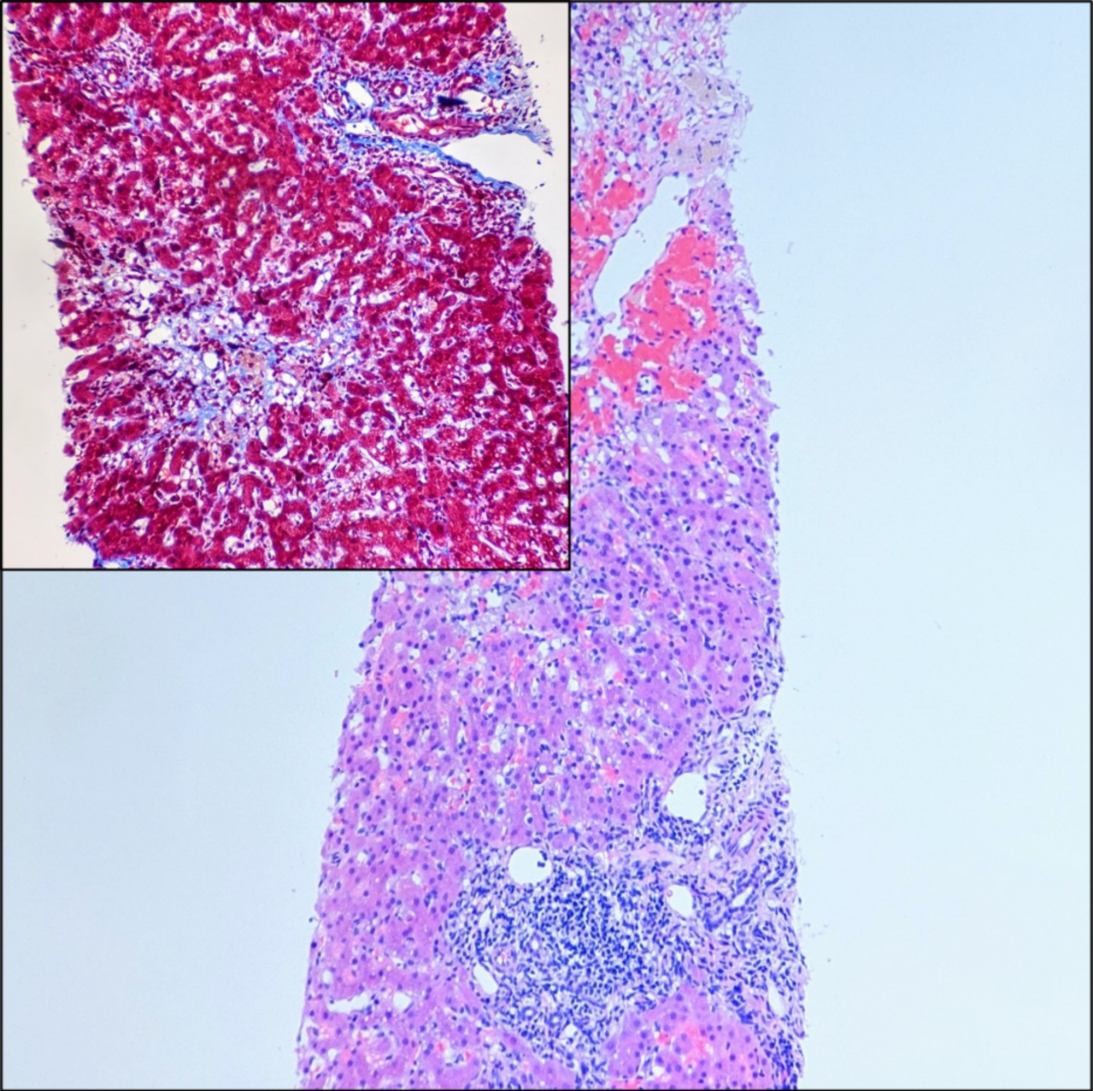
Liver parenchyma showing mixed portal inflammation consisting of lymphocytes, eosinophils, neutrophils and rare plasma cells with scattered lobular activity and acidophil bodies suggestive of hepatocellular injury. Areas of pericentral hepatocyte dropout are suggestive of vascular insult in zone 3 (hematoxylin-eosin stain, ×40). Inset shows the lack of periportal or pericentral fibrosis. It also highlights the pale blue areas, representing trichrome staining around the central area, a feature of hepatocellular dropout and injury as opposed to the bright blue areas with fibrosis (trichrome stain, ×100).

Five weeks after the transplant, she was admitted to the hospital for intractable nausea, vomiting and inability to keep down her immunosuppression medications. Her LFTs were elevated raising concerns for rejection. As a result, serologic workup was repeated and came back negative. Ultrasound of the abdomen with Doppler showed no pathology and demonstrated normal flow in the hepatic vessels. Her immunosuppression drug levels were noted to be within therapeutic range on admission. A liver biopsy was performed and showed mixed, predominantly portal inflammation, hepatocellular injury and vascular insult. It did not demonstrate rejection or fibrosis (Figure 2). Acute rejection of allograft liver is defined by a scoring system called the Rejection Activity Index, set by the World Gastroenterology Consensus Document. It provides a score for the degree of portal, ductal and venous endothelial inflammation, lymphocyte infiltration, edema, reactive or degenerative changes and necrosis seen in the biopsy. The degree of portal inflammation, hepatocellular injury and vascular insult seen in the liver biopsy was mild and did not meet the Rejection Activity Index's score for acute rejection. 

Within a week of admission, she developed worsening anemia, thrombocytopenia and acute kidney injury. Hemolysis labs were consistent with hemolytic anemia with an elevated lactate dehydrogenase, low haptoglobin, elevated indirect bilirubin and schistocytes on peripheral smear. Due to evidence of microangiopathic hemolysis and thrombocytopenia suggestive of thrombotic microangiopathy, plasmapheresis was initiated, tacrolimus was held and the patient was placed on steroids instead.

Despite three sessions of plasmapheresis and withdrawal of CNI, the patient’s labs did not improve. ADAMTS13 activity was 91%. CH50 complement was low. Due to lack of response to plasmapheresis, this was not believed to be TTP and with lack of improvement despite withdrawing CNI, CNI-induced TMA seemed less likely. The diagnosis of aHUS was made, and the patient was started on eculizumab. She was started on an induction dose of 900 mg weekly for four weeks and then maintenance with 1,200 mg at week 5 and every two weeks thereafter.

Encapsulated organism prophylaxis was followed. Meningococcal vaccination was given. After the first dose of eculizumab, the patient's hemolysis parameters began to improve and creatinine took about three weeks to return to baseline of 0.7-0.9.

Eculizumab was stopped after three months of therapy due to difficulties getting it covered by insurance. Fortunately, her renal function as well as hemoglobin and platelets remained stable up to one year of follow-up post-treatment. However, on treatment she did not experience any of the commonly seen side effects of eculizumab such as headache, hypertension, diarrhea, or leukopenia or meningitis.

## Discussion

aHUS is a clinical diagnosis based on the classic triad of microangiopathic hemolytic anemia, thrombocytopenia and acute kidney injury. Diagnosis of aHUS relies on (1) no associated disease; (2) no criteria for Shigatoxin-HUS (stool culture and polymerase chain reaction for Shiga-toxins; serology for anti-lipopolysaccharides antibodies); (3) no criteria for TTP (serum ADAMTS 13 activity > 10%) [1]. Workup should include complement and genetic testing, although the diagnosis cannot be excluded in the setting of normal complement levels or genetic workup [1-4]. The minimum set of genes that should be screened includes CFH, CD46, CFI, C3, CFB, THBD, CFHR1, CFHR5 and DGKE. ADAMTS13 activity is critical to exclude TTP [2].

Once a diagnosis of aHUS is suspected, possible treatment options include plasma exchange, eculizumab and renal transplant. Eculizumab, a monoclonal antibody that prevents c5 cleavage and indirectly inhibits the formation of the membrane attack complex, is being used to treat aHUS [5]. Early initiation of eculizumab at the time of diagnosis has been associated with significantly improved clinical outcomes, reversal of organ damage and better quality of life [6]. Current dosing recommendation for adults is IV induction with 900 mg weekly for four doses and maintenance with 1,200 mg at week 5 followed by 1,200 mg every two weeks. There are two alternative dosing regimens suggested for minimal dosing and discontinuation of treatment, with no supporting evidence. Minimal dosing is achieved with a goal of CH50 or AH50 < 10%, with a goal eculizumab trough level 50-100 µg/ml. Clinical response and risk of recurrence are the mainstays of withdrawing eculizumab. If eculizumab is to be discontinued, it is recommended to have frequent laboratory monitoring for complement monitoring. Currently, no consensus exists for dose tapering, duration of treatment or frequency and duration of monitoring laboratory parameters on withdrawal of drug. Current guidelines do not recommend cessation of treatment in the setting of concurrent illness as it would increase the risk of aHUS relapse, unless there is suspected or proven infection with an encapsulated organism [2].

Unfortunately, access to eculizumab is limited due to its high cost estimated at an annual $700,000 for one patient per America's Health Insurance Plans estimates of high priced specialty medications. Plasma therapy can be used instead, but there is a risk of suboptimal therapeutic outcome. Plasma therapy is also indicated in emergent cases of severe thrombotic microangiopathy or clinical suspicion of TTP until ADAMTS13 activity is shown to be >10%. Kidney transplantation should be delayed till six months after starting dialysis because of the possibility of renal recovery with eculizumab. If a patient is to require renal transplant, eculizumab may be used prophylactically based on recurrence risk. Liver transplant can be considered in patients who have had prior renal transplants due to liver-derived complement protein abnormalities, who have recurrent renal failure despite treatment with eculizumab [2].

## Conclusions

Due to the high rates of mortality and risk of progression to end-stage renal disease, this case report highlights the effectiveness of a relatively new drug, eculizumab, in treating cases of aHUS that are otherwise not responsive to older conventional modalities of treatment such as withdrawal of CNI or plasmapheresis. It is important to highlight that the patient achieved a quick and favorable clinical response to eculizumab, despite receiving treatment for a shorter than recommended period of three months. The patient maintained clinical remission for at least one year, after which she had relocated and was lost to follow-up. As no consensus exits within current guidelines as to duration and discontinuation of treatment, this case adds more evidence to this aspect. Further studies are needed to effectively establish a safe and effective duration of treatment. 
